# Synthesis, antibacterial and anticancer activity, and docking study of aminoguanidines containing an alkynyl moiety

**DOI:** 10.1080/14756366.2019.1702654

**Published:** 2019-12-18

**Authors:** Xianqing Deng, Mingxia Song

**Affiliations:** Basic Medical and Pharmacy College, Jinggangshan University, Ji’an, China

**Keywords:** Alkynyl, aminoguanidine, antibacterial activity, anticancer, docking

## Abstract

Two series of aminoguanidines containing an alkynyl moiety were designed, synthesised, and screened for antibacterial and anticancer activities. Generally, the series **3a–3j** with a 1,2-diphenylethyne exhibited better antibacterial activity than the other series (**6a–6k**) holding 1,4-diphenylbuta-1,3-diyne moiety antibacterial activity. Most compounds in series **3a–3j** showed potent growth inhibition against the tested bacterial strains, with minimum inhibitory concentration (MIC) values in the range 0.25–8 µg/mL. Compound **3g** demonstrated rapid and persistent bactericidal activity at 2 × MIC. The resistance study revealed that resistance of the tested bacteria towards **3g** is not easily developed. Molecular docking studies revealed that compounds **3g** and **6e** bind strongly to the LpxC and FabH enzymes. Moreover, excellent activity of selected compounds against the growth of cancer cell lines A549 and SGC7901 was also observed, with IC_50_ values in the range 0.30–4.57 µg/mL. These findings indicate that compounds containing the aminoguanidine moiety are promising candidates for the development of new antibacterial and anticancer agents.

## Introduction

Multidrug-resistant (MDR) Gram-negative bacterial strains have arisen against all antibiotics in clinical use. Infections caused by these MDR bacteria threaten global public health^1^^,^^2^ and are associated with high mortality rates. New antibacterial drugs with novel chemical scaffolds and targets are urgently required to combat infections due to drug-resistant strains^3^^,^^4^.

One of the validated antibiotic targets against Gram-negative bacteria is UDP-3-*O*-(*R*-3-hydroxymyristoyl)-*N*-acetylglucosamine deacetylase (LpxC), an essential enzyme catalysing the first committed step in the lipid A biosynthetic pathway. Because there is no gene homology in humans, inhibiting LpxC could result in the death of bacteria without causing side effects in the body^5^. Based upon these facts, LpxC has become a promising target for developing novel therapeutics against MDR Gram-negative pathogens^6–8^.

Studies of the enzymatic mechanism underlying LpxC have identified a hydrophobic tunnel that binds a myristate fatty acyl chain of the natural substrate and leads to a Zn^2+^ active site responsible for deacetylation^9^^,^^10^. Since the discovery of L-161240 as the first LpxC inhibitor in the 1990s^11^, many LpxC inhibitors have been reported as antibacterial agents^6–8^. Most LpxC inhibitors share common structural features that mimic the natural Zn^2+^-binding substrate: (1) a hydroxamate head group, (2) a central linker, and (3) a lipophilic tail^12^. Among the well-characterised compounds, threonyl-hydroxamate derivatives, such as CHIR-12 and LPC-009, are representative LpxC inhibitors^13–15^. The hydroxamate group of these compounds occupies the active site, and their diphenyl acetylene or phenyl-diyne group penetrates the hydrophobic passage^16^^,^^17^.

Aminoguanidine is functional group with a high polarity and capacity for hydrogen bonding with many critical amino acid residues as well as metal ions. Many aminoguanidine derivatives exhibit antitumor activity *via* the formation of complexes with metal ions. In our previous work, some aminoguanidine derivatives were reported as potent antibacterial agents against Gram-positive bacteria and Gram-negative bacteria including MDR clinical isolates^18–20^. The binding of these compounds involves a specific interaction with the *β*-ketoacyl-acyl carrier protein synthase III (FabH) enzyme^20^. By analysing the SAR of these compounds, it can be concluded that aminoguanidine combined with a hydrophobic moiety in the form of azomethine imine is the common structural requirement for their antibacterial activity.

**Figure 1.**
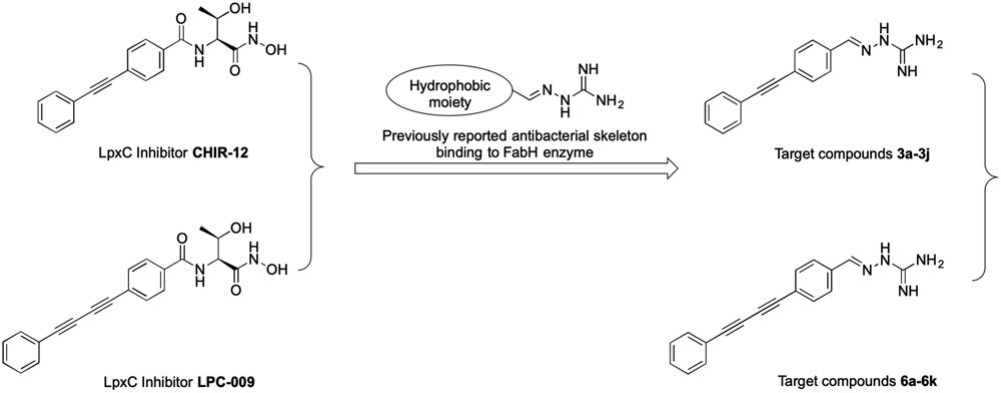
The design of target compounds.

In this contribution, we built upon the above observations by replacing the threonyl-hydroxamate group in the lead compounds CHIR-12 and LPC-009 with aminoguanidine (Figure 1), yielding two series of aminoguanidines **3a–3j** and **6a–6k**. We anticipated that this design would promote binding with both FabH and LpxC, resulting in high and broad-spectrum antibacterial activity.

All the synthesised compounds were characterised by ^1^H-NMR, ^13^C-NMR, and high-resolution mass spectrometry, then evaluated for their antibacterial activity against 12 bacterial strains. To further characterise the antibacterial effects of compound **3g**, the propensity for the development of bacterial resistance was determined and a bactericidal time-kill assay was carried out. Molecular docking studies of representative compounds **3g** and **6e** with LpxC and FabH were performed to understand the binding mechanism. Considering the reported anticancer activity of numerous compounds containing the guanidine moiety^21^^,^^22^, the anticancer activity was also evaluated for some selected compounds against two cancer cell lines (A549 and SGC7901). In this work, some aminoguanidines were discovered with promising antibacterial and anticancer activities.

## Materials and methods

### Instruments and reagents

All the reagents and solvents were purchased from Aladdin (Shanghai, China) or Sinopharm Chemical Reagent Co. Ltd. (Shanghai, China), and were used as received. Melting points were determined in open capillary tubes and are uncorrected. Reaction courses were monitored by thin-layer chromatography (TLC) on silica gel-precoated F_254_ plates (Merck, Darmstadt, Germany). Developed plates were examined with UV lamps (254 nm). Nuclear magnetic resonance spectroscopy was performed on an AV-400 spectrometer (Bruker, Zurich, Switzerland) operating at 400 MHz for ^1^H and 100 MHz for ^13^C and using DMSO-*d_6_* as the solvent and tetramethylsilane as the internal standard. Matrix-assisted laser desorption/ionization time-of-flight mass spectrometry (MALDI-MS) experiments were performed on a Bruker ultrafleXtreme MALDI-TOF/TOF mass spectrometer (Bruker Daltonik GmbH, Leipzig, Germany) equipped with a smartbeam II laser (1000 Hz).

### Chemistry

#### Synthesis of 4-(substituted-phenylethynyl)benzaldehyde (2a–2j)

To a stirred solution of 4-ethynylbenzaldehyde (6.15 mmol, 1 eq) in THF (8 ml) was added substituted-iodobenzenes (6.76 mmol, 1 eq), TEA (0.4 ml), CuI (80 mg, 0.42 mmol, 0.07 eq) and Pd(PPh_3_)Cl_2_ (0.18 mmol, 0.03 eq) under N_2_. After stirring for 3 h at 45 °C, the resulting mixture was concentrated under vacuum. The residue was applied onto a silica gel column eluted with 1**–**4% ethyl acetate in petroleum ether to afford 4-(substituted-phenylethynyl)benzaldehyde **(2a–2j)** as a light yellow solid.

#### Synthesis of 2-(4-(substituted -phenylethynyl)benzylidene)hydrazine-1-carboximidamide (3a–3j)

To a stirred solution of hydrazinecarboximidamide carbonate (5.04 mmol, 1.3 eq) in water (8 ml) was added NaOAc (5.04 mmol, 1.3 eq). After stirring for 0.5 h at room temperature, a mixture of 4-(substituted-phenylethynyl)benzaldehyde (**2a–2j**) (3.88 mmol, 1 eq) in EtOH (8 ml) was added. Then the resulting solution was stirred at 70 °C for 6 h. The reaction mixture was diluted with water (16 ml) and then cooled to room temperature. After stirred for 3 h, large amount of solids was precipitated. The solids were collected by filtration, washed with EtOH (2 × 0.8 ml), and then dried in an oven under reduced pressure to afford 2-(4-(substituted-phenylethynyl)benzylidene)hydrazine-1-carboximidamide (**3a–3j**) as a light yellow solid.

#### 2-(4-(Phenylethynyl)benzylidene)hydrazine-1-carboximidamide (3a)

Light yellow solid, m.p. 225–226 °C, yield 88%. ^1^H-NMR (DMSO-*d_6_*, 400 MHz): *δ* 5.65 (s, 2H, NH_2_), 6.04 (s, 2H, NH), 7.42–7.57 (m, 5H, Ar-H), 7.50 (d, 2H, *J* = 8.2 Hz, Ar-H), 7.72 (d, 2H, *J* = 8.2 Hz, Ar-H), 7.99 (s, 1H, N=CH). ^13^C-NMR (DMSO-*d_6_*, 100 MHz): *δ* 160.96, 142.06, 137.48, 131.43, 131.33, 128.80, 128.75, 126.32, 122.43, 120.88, 90.09, 89.84. MS *m/z* 263 (M + 1). ESI-HRMS calcd for C16H15N4^+^ ([M + H]^+^) 263.1291; found: 263.1287.

#### 2-(4-((2-Fluorophenyl)ethynyl)benzylidene)hydrazine-1-carboximidamide (3b)

Light yellow solid, m.p. 189–190 °C, yield 75%. ^1^H-NMR (DMSO-*d_6_*, 400 MHz): *δ* 5.63 (s, 2H, NH_2_), 6.02 (s, 2H, NH_2_), 7.26–7.65 (m, 6H, Ar-H), 7.72 (d, 2H, *J* = 7.7 Hz, Ar-H), 7.99 (s, 1H, N=CH). ^13^C-NMR (DMSO-*d_6_*, 100 MHz): *δ* 162.20 (d, ^1^*J*_c-f_ = 247.72 Hz), 161.51, 142.38, 138.36, 133.86, 131.93, 131.43 (d, ^3^*J*_c-f_ = 7.92 Hz), 126.79, 125.32 (d, ^4^*J*_c-f_ = 3.4 Hz), 120.77, 116.22 (d, ^2^*J*_c-f_ = 20.8 Hz), 111.25 (d, ^2^*J*_c-f_ = 15.4 Hz), 95.25, 83.68. MS *m/z* 281 (M + 1). ESI-HRMS calcd for C16H14FN4^+^ ([M + H]^+^) 281.1197; found: 281.1193.

#### 2-(4-((3-Fluorophenyl)ethynyl)benzylidene)hydrazine-1-carboximidamide (3c)

Light yellow solid, m.p. 223–224 °C, yield 76%. ^1^H-NMR (DMSO-*d_6_*, 400 MHz): *δ* 5.68 (s, 2H, NH_2_), 6.06 (s, 2H, NH_2_), 7.21–7.48 (m, 4H, Ar-H), 7.51 (d, 2H, *J* = 8.4 Hz, Ar-H), 7.74 (d, 2H, *J* = 8.4 Hz, Ar-H), 8.00 (s, 1H, N=CH). ^13^C-NMR (DMSO-*d_6_*, 100 MHz): *δ* 161.92 (d, ^1^*J*_c-f_ = 243.1 Hz), 161.00, 141.98, 137.79, 131.56, 130.89 (d, ^3^*J*_c-f_ = 8.9 Hz), 127.71 (d, ^4^*J*_c-f_ = 2.7 Hz), 126.33, 124.42 (d, ^3^*J*_c-f_ = 9.5 Hz), 120.39, 117.87 (d, ^2^*J*_c-f_ = 22.7 Hz), 115.96 (d, ^2^*J*_c-f_ = 20.9 Hz), 90.80, 88.78. MS *m/z* 281 (M + 1). ESI-HRMS calcd for C16H14FN4^+^ ([M + H]^+^) 281.1197; found: 281.1196.

#### 2-(4-((4-Fluorophenyl)ethynyl)benzylidene)hydrazine-1-carboximidamide (3d)

Light yellow solid, m.p. 238–239 °C, yield 69%. ^1^H-NMR (DMSO-*d_6_*, 400 MHz): *δ* 5.62 (s, 2H, NH_2_), 6.01 (s, 2H, NH_2_), 7.26–7.64 (m, 4H, Ar-H), 7.49 (d, 2H, *J* = 8.3 Hz, Ar-H), 7.72 (d, 2H, *J* = 8.3 Hz, Ar-H), 7.99 (s, 1H, N=CH). ^13^C-NMR (DMSO-*d_6_*, 100 MHz): *δ* 161.99 (d, ^1^*J*_c-f_ = 246 Hz), 160.99, 142.02, 137.53, 133.65 (d, ^3^*J*_c-f_ = 8.5 Hz), 131.40, 126.30, 120.74, 118.92, 116.05 (d, ^2^*J*_c-f_ = 22.0 Hz), 89.56, 89.02. MS *m/z* 281 (M + 1). ESI-HRMS calcd for C16H14FN4^+^ ([M + H]^+^) 281.1197; found: 281.1199.

#### 2-(4-((2-Chlorophenyl)ethynyl)benzylidene)hydrazine-1-carboximidamide (3e)

Light yellow solid, m.p. 200–203 °C, yield 81%. ^1^H-NMR (DMSO-*d_6_*, 400 MHz): *δ* 5.71 (s, 2H, NH_2_), 6.07 (s, 2H, NH_2_), 7.38–7.69 (m, 4H, Ar-H), 7.54 (d, 2H, *J* = 8.3 Hz, Ar-H), 7.75 (d, 2H, *J* = 8.3 Hz, Ar-H), 8.01(s, 1H, N=CH). ^1^^3^C-NMR (DMSO-*d_6_*, 100 MHz): *δ* 161.01, 141.97, 137.91, 134.53, 133.31, 131.52, 130.28, 129.42, 127.42, 126.37, 122.14, 120.38, 94.86, 86.73. MS *m/z* 297 (M + 1). ESI-HRMS calcd for C16H14ClN4^+^ ([M + H]^+^) 297.0902; found: 297.0906.

#### 2-(4-((3-Chlorophenyl)ethynyl)benzylidene)hydrazine-1-carboximidamide (3f)

Light yellow solid, m.p. 213–214 °C, yield 80%. ^1^H-NMR (DMSO-*d_6_*, 400 MHz): *δ* 5.62 (s, 2H, NH_2_), 6.02 (s, 2H, NH_2_), 7.44–7.65 (m, 6H, Ar-H), 7.73 (d, 2H, *J* = 8.4 Hz, Ar-H), 7.99 (s, 1H, N=CH). ^13^C-NMR (DMSO-*d_6_*, 100 MHz): *δ* 161.06, 141.90, 137.88, 133.35, 131.56, 130.74, 130.66, 129.99, 128.79, 126.30, 124.44, 120.29, 91.17, 88.55. MS *m/z* 297 (M + 1). ESI-HRMS calcd for C16H14ClN4^+^ ([M + H]^+^) 297.0902; found: 297.0900.

#### 2-(4-((4-Chlorophenyl)ethynyl)benzylidene)hydrazine-1-carboximidamide (3g)

Light yellow solid, m.p. 232–234 °C, yield 77%. ^1^H-NMR (DMSO-*d_6_*, 400 MHz): ^1^5.73 (s, 2H, NH_2_), 6.08 (s, 2H, NH_2_), 7.48–7.59 (m, 6H, Ar-H), 7.73 (d, 2H, *J* = 8.3 Hz, Ar-H), 8.00 (s, 1H, N=CH). ^13^C-NMR (DMSO-*d_6_*, 100 MHz): *δ* 160.88, 142.03, 137.62, 133.41, 133.03, 131.48, 128.95, 126.34, 121.32, 120.59, 90.91, 88.95. MS *m/z* 297 (M + 1). ESI-HRMS calcd for C16H14ClN4^+^ ([M + H]^+^) 297.0902; found: 297.0908.

#### 2-(4-((2-Bromophenyl)ethynyl)benzylidene)hydrazine-1-carboximidamide (3h)

Light yellow solid, m.p(0).188–190 °C, yield 65%. ^1^H-NMR (DMSO-*d*_6_, 400 MHz): *δ* 5.71 (s, 2H, NH_2_), 6.07 (s, 2H, NH_2_), 7.33–7.68 (m, 4H, Ar-H), 7.53 (d, 2H, *J* = 8.2 Hz, Ar-H), 7.75 (d, 2H, *J* = 8.2 Hz, Ar-H), 8.02 (s, 1H, N=CH). ^13^C-NMR (DMSO-*d_6_*, 100 MHz): *δ* 160.98, 141.99, 137.88, 133.30, 132.51, 131.45, 130.37, 127.87, 126.35, 124.69, 124.31, 120.42, 94.16, 88.64. MS *m/z* 341 (M + 1). ESI-HRMS calcd for C16H14BrN4^+^ ([M + H]^+^) 341.0396; found: 341.0397.

#### 2-(4-((3-Bromophenyl)ethynyl)benzylidene)hydrazine-1-carboximidamide (3i)

Light yellow solid, m.p. 223–225 °C, yield 73%. ^1^H-NMR (DMSO-*d_6_*, 400 MHz): *δ* 5.66 (s, 2H, NH_2_), 6.04 (s, 2H, NH_2_), 7.36–7.78 (m, 4H, Ar-H), 7.54 (d, 2H, *J* = 8.3 Hz, Ar-H), 7.73 (d, 2H, *J* = 8.3 Hz, Ar-H), 8.00 (s, 1H, N=CH). ^13^C-NMR (DMSO-*d_6_*, 100 MHz): *δ* 161.04, 141.91, 137.85, 133.55, 131.64, 131.56, 130.83, 130.30, 126.30, 124.69, 121.74, 120.31, 91.26, 88.46. MS *m/z* 341 (M + 1). ESI-HRMS calcd for C16H14BrN4^+^ ([M + H]^+^) 341.0396; found: 341.0391.

#### 2-(4-((4-Bromophenyl)ethynyl)benzylidene)hydrazine-1-carboximidamide (3j)

Light yellow solid, m.p. 242–243 °C, yield 70%. ^1^H-NMR (DMSO-*d_6_*, 400 MHz): *δ* 5.65 (s, 2H, NH_2_), 6.03 (s, 2H, NH_2_), 7.49–7.52 (m, 4H, Ar-H), 7.64 (d, 2H, *J* = 8.4 Hz, Ar-H), 7.72 (d, 2H, *J* = 8.4 Hz, Ar-H), 7.99 (s, 1H, N=CH). 13C-NMR (DMSO-d_6_, 100 MHz): *δ* 160.98, 141.96, 137.71, 133.22, 131.84, 131.47, 126.31, 122.09, 121.67, 120.51, 91.06, 89.02. MS *m/z* 341 (M + 1). ESI-HRMS calcd for C16H14BrN4^+^ ([M + H]^+^) 341.0396; found: 341.0396.

#### Synthesis of 4-(iodoethynyl)benzaldehyde (4)

Ethynylbenzaldehyde (0.140 g, 1.076 mmol) was dissolved in acetone (10 ml) and AgNO_3_ (0.055 g, 0.323 mmol) was added. After 0.5 h Et_2_O (30 ml) and *N*-Iodosuccinimide (0.242 g, 1.076 mmol) were added. The mixture was stirred for 12 h after which time it was filtered and washed with ice-cold H_2_O (30 ml). The organic layer was separated. The aqueous layer was washed with Et_2_O (2 × 5 ml) and the combined organic parts were dried with Na_2_SO_4_. The solvent was removed under oil-pump vacuum and the residue was purified by chromatography on silica gel (20 cm; hexane/CH_2_Cl_2_ v/v, 1:1) to give 4-(iodoethynyl)benzaldehyde (**4**) as a yellow powder (Yield = 60%)^23^.

#### Synthesis of 4-((substituted-phenyl)buta-1,3-diyn-1-yl)benzaldehyde (5a–5k)

To a stirred solution of 4-(iodoethynyl)benzaldehyde (800 mg, 3.12 mmol, 1 eq) in THF (8 ml) was added substituted-ethynylbenzene (3.32 mmol, 1. 07 eq), TEA (0.4 ml), CuI (42 mg, 0.22 mmol, 0.07 eq) and Pd(PPh_3_)Cl_2_ (65 mg, 0. 09 mmol, 0.03 eq) under N_2_. After stirring overnight at room temperature, the resulting mixture was concentrated under vacuum. The residue was applied onto a silica gel column eluted with 1**–**2% ethyl acetate in petroleum ether to afford a yellow solid.

#### Synthesis of 2-(4-((substituted-phenyl)buta-1,3-diyn-1-yl)benzylidene)hydrazine-1-carboximidamide (6a–6k)

To a stirred solution of hydrazinecarboximidamide carbonate (124.03 mg, 0.91 mmol, 1.4 eq) in water (5 ml) was added NaOAc (74.78 mg, 0.91 mmol, 1.4 eq). After stirring for 0.5 h at room temperature, a mixture of 4-((substituted-phenyl)buta-1,3-diyn-1-yl)benzaldehyde (0.65 mmol, 1 eq) in EtOH (5 ml) was added. Then the resulting solution was refluxed overnight. The reaction mixture was diluted with water (15 ml) and then cooled to room temperature. After stirred for 3 h, large amount of solids was precipitated. The solids were collected by filtration, washed with EtOH (2**–**0.5 ml), and then dried in an oven under reduced pressure to afford an off white solid.

#### 2-(4-(Phenylbuta-1,3-diyn-1-yl)benzylidene)hydrazine-1-carboximidamide (6a)

Off white solid, m.p. 209–212 °C, yield 64%. ^1^H-NMR (DMSO-*d_6_*, 400 MHz): *δ* 5.66 (s, 2H, NH_2_), 6.05 (s, 2H, NH_2_), 7.43–7.63 (m, 5H, Ar-H), 7.54 (d, 2H, *J* = 8.4 Hz, Ar-H), 7.73 (d, 2H, *J* = 8.4 Hz, Ar-H), 7.98 (s, 1H, N=CH).^13^C-NMR (DMSO-*d_6_*, 100 MHz): *δ* 161.17, 141.63, 138.70, 132.48, 132.37, 129.96, 128.94, 126.30, 120.54, 118.69, 82.48, 82.26, 74.24, 73.76. MS *m/z* 287 (M + 1). ESI-HRMS calcd for C18H15N4^+^ ([M + H]^+^) 287.1291; found: 287.1286.

#### 2-(4-((2-Fluorophenyl)buta-1,3-diyn-1-yl)benzylidene)hydrazine-1-carboximidamide (6 b)

Off white solid, m.p. 218–220 °C, yield 76%. ^1^H-NMR (DMSO-*d_6_*, 400 MHz): *δ* 5.83 (s, 2H, NH_2_), 6.17 (s, 2H, NH_2_), 7.27–7.71 (m, 6H, Ar-H), 7.75 (d, 2H, *J* = 8.4 Hz, Ar-H), 7.99 (s, 1H, N=CH). ^13^C-NMR (DMSO-*d_6_*, 100 MHz): *δ* 162.97 (d, ^1^*J*_c-f_ = 249.6 Hz), 160.90, 141.72, 138.72, 134.35, 132.57, 132.25 (d, ^3^*J*_c-f_ = 8.0 Hz), 126.37, 125.05 (d, ^4^*J*_c-f_ = 3.5 Hz), 118.50, 115.92 (d, ^2^*J*_c-f_ = 20.0 Hz), 109.14 (d, ^2^*J*_c-f_ = 15.2 Hz), 83.64, 78.40, 75.43, 73.85. MS *m/z* 305 (M + 1). ESI-HRMS calcd for C18H14FN4^+^ ([M + H]^+^) 305.1197; found: 305.1195.

#### 2-(4-((3-Fluorophenyl)buta-1,3-diyn-1-yl)benzylidene)hydrazine-1-carboximidamide (6c)

Off white solid, m.p. 186–190 °C, yield 69%. ^1^H-NMR (DMSO-*d_6_*, 400 MHz): *δ* 5.84 (s, 2H, NH_2_), 6.13 (s, 2H, NH_2_), 7.34–7.55 (m, 4H, Ar-H), 7.58 (d, 2H, *J* = 8.4 Hz, Ar-H), 7.74 (d, 2H, *J* = 8.4 Hz, Ar-H), 7.99 (s, 1H, N=CH). ^13^C-NMR (DMSO-*d_6_*, 100 MHz): *δ* 161.79 (d, ^1^*J*_c-f_ = 243.8 Hz), 161.08, 141.59, 138.82, 132.58, 131.14 (d, ^3^*J*_c-f_ = 8.8 Hz), 128.86, 126.35, 122.52 (d, ^3^*J*_c-f_ = 9.8 Hz), 118.99 (d, ^2^*J*_c-f_ = 23.2 Hz), 118.48, 117.43 (d, ^2^*J*_c-f_ = 20.8 Hz), 83.12, 80.78, 74.58, 73.96. MS *m/z* 305 (M + 1). ESI-HRMS calcd for C18H14FN4^+^ ([M + H]^+^) 305.1197; found: 305.1199.

#### 2-(4-((4-Fluorophenyl)buta-1,3-diyn-1-yl)benzylidene)hydrazine-1-carboximidamide (6d)

Off white solid, m.p. 206–210 °C, yield 78%. ^1^H-NMR (DMSO-*d_6_*, 400 MHz): *δ* 5.91 (s, 2H, NH_2_), 6.22 (s, 2H, NH_2_), 7.27–7.75 (m, 4H, Ar-H), 7.54 (d, 2H, *J* = 8.4 Hz, Ar-H), 7.74 (d, 2H, *J* = 8.4 Hz, Ar-H), 7.99 (s, 1H, N=CH). ^13^C-NMR (DMSO-*d_6_*, 100 MHz): *δ* 162.70 (d, ^1^*J*_c-f_ = 248.3 Hz), 160.76, 141.86, 138.45, 134.95 (d, ^3^*J*_c-f_ = 8.8 Hz), 132.51, 126.42, 118.90, 117.05(d, ^4^*J*_c-f_ = 3.3 Hz), 116.34 (d, ^2^*J*_c-f_ = 22.2 Hz), 82.36, 81.29, 74.26, 73.54. MS *m/z* 305 (M + 1). ESI-HRMS calcd for C18H15FN4^+^ ([M + H]^+^) 305.1197; found: 305.1189.

#### 2-(4-((2-Chlorophenyl)buta-1,3-diyn-1-yl)benzylidene)hydrazine-1-carboximidamide (6e)

Off white solid, m.p. 207–210 °C, yield 75%. ^1^H-NMR (DMSO-*d_6_*, 400 MHz): *δ* 5.95 (s, 2H, NH_2_), 6.23 (s, 2H, NH_2_), 7.38–7.76 (m, 8H, Ar-H), 8.00 (s, 1H, N=CH). ^13^C-NMR (DMSO-*d_6_*, 100 MHz): *δ* 161.31, 142.23, 139.12, 136.04, 135.02, 133.06, 131.85, 130.02, 128.01, 126.86, 121.01, 119.05, 84.51, 79.03, 78.81, 74.43. MS *m/z* 321(M + 1). ESI-HRMS calcd for C18H14ClN4^+^ ([M + H]^+^) 321.0902; found: 321.0906.

#### 2-(4-((3-Chlorophenyl)buta-1,3-diyn-1-yl)benzylidene)hydrazine-1-carboximidamide (6f)

Off white solid, m.p. 198–201 °C, yield 81%. ^1^H-NMR (DMSO-*d_6_*, 400 MHz): *δ* 5.83 (s, 2H, NH_2_), 6.12 (s, 2H, NH_2_), 7.45–7.50 (m, 8H, Ar-H), 7.99 (s, 1H, N=CH). ^13^C-NMR (DMSO-*d_6_*, 100 MHz): *δ* 161.09, 141.57, 138.83, 133.46, 132.57, 131.67, 131.15, 130.78, 130.08, 126.33, 122.58, 118.44, 83.23, 80.58, 74.88, 73.96. MS *m/z* 321 (M + 1). ESI-HRMS calcd for C18H14ClN4^+^ ([M + H]^+^) 321.0902; found: 321.0901.

#### 2-(4-((4-Chlorophenyl)buta-1,3-diyn-1-yl)benzylidene)hydrazine-1-carboximidamide (6g)

Off white solid, m.p. 202–205 °C, yield 45%. ^1^H-NMR (DMSO-*d_6_*, 400 MHz): *δ* 5.92 (s, 2H, NH_2_), 6.21 (s, 2H, NH_2_), 7.51–7.56 (m, 4H, Ar-H), 7.64 (d, 2H, *J* = 8.3 Hz, Ar-H), 7.74 (d, 2H, *J* = 8.3 Hz, Ar-H), 7.99 (s, 1H, N=CH). ^13^C-NMR (DMSO-*d_6_*, 100 MHz): *δ* 159.84, 142.21, 137.82, 134.86, 134.18, 132.62, 129.19, 126.74, 119.44, 82.83, 81.22, 74.69, 74.33. MS *m/z* 321 (M + 1). ESI-HRMS calcd for C18H14ClN4^+^ ([M + H]^+^) 321.0902; found: 321.0909.

#### 2-(4-((2-Bromophenyl)buta-1,3-diyn-1-yl)benzylidene)hydrazine-1-carboximidamide (6h)

Off white solid, m.p. 217–219 °C, yield 72%. ^1^H-NMR (DMSO-*d_6_*, 400 MHz): *δ* 5.69 (s, 2H, NH_2_), 6.06 (s, 2H, NH_2_), 7.39–7.78 (m, 6H, Ar-H), 7.58 (d, 2H, *J* = 8.4 Hz, Ar-H), 7.99 (s, 1H, N=CH). ^13^C-NMR (DMSO-*d_6_*, 100 MHz): *δ* 161.68, 142.02, 139.40, 135.20, 133.14, 133.05, 131.99, 128.50, 126.78, 125.79, 123.23, 118.82, 84.56, 80.73, 78.13, 74.41. MS *m/z* 365 (M + 1). ESI-HRMS calcd for C18H14ClN4^+^ ([M + H]^+^) 365.0396; found: 365.0394.

#### 2-(4-((3-Bromophenyl)buta-1,3-diyn-1-yl)benzylidene)hydrazine-1-carboximidamide (6i)

Off white solid, m.p. 196–198 °C, yield 68%. ^1^H-NMR (DMSO-*d_6_*, 400 MHz): *δ* 5.95 (s, 2H, NH_2_), 6.24 (s, 2H, NH_2_), 7.38–7.83 (m, 4H, Ar-H), 7.56 (d, 2H, *J* = 8.4 Hz, Ar-H), 7.75 (d, 2H, *J* = 8.4 Hz, Ar-H), 7.99 (s, 1H, N=CH). ^13^C-NMR (DMSO-*d_6_*, 100 MHz): *δ* 160.70, 141.82, 138.57, 134.45, 132.94, 132.58, 131.50, 130.93, 126.43, 122.83, 121.81, 118.66, 83.21, 80.51, 74.95, 74.04. MS *m/z* 365 (M + 1). ESI-HRMS calcd for C18H14ClN4^+^ ([M + H]^+^) 365.0396; found: 365.0391.

#### 2-(4-((4-Bromophenyl)buta-1,3-diyn-1-yl)benzylidene)hydrazine-1-carboximidamide (6j)

Off white solid, m.p. 227–230 °C, yield 50%. ^1^H-NMR (DMSO-*d_6_*, 400 MHz): *δ* 5.93 (s, 2H, NH_2_), 6.22 (s, 2H, NH_2_), 7.55–7.57 (m, 4H, Ar-H), 7.65 (d, 2H, *J* = 8.4 Hz, Ar-H), 7.74 (d, 2H, *J* = 8.4 Hz, Ar-H), 8.00 (s, 1H, N=CH). ^13^C-NMR (DMSO-*d_6_*, 100 MHz): *δ* 161.12, 142.37, 138.92, 134.69, 132.99, 132.49, 126.92, 124.05, 120.25, 119.27, 83.47, 81.66, 75.30, 74.64. MS *m/z* 365 (M + 1). ESI-HRMS calcd for C18H14ClN4^+^ ([M + H]^+^) 365.0396; found: 365.0395.

#### 2-(4-(m-Tolylbuta-1,3-diyn-1-yl)benzylidene)hydrazine-1-carboximidamide (6k)

Off white solid, m.p. 202–204 °C, yield 63%. ^1^H-NMR (DMSO-*d_6_*, 400 MHz): *δ* 2.32 (s, 3H, CH_3_), 5.72 (s, 2H, NH_2_), 6.09 (s, 2H, NH_2_), 7.29–7.43 (m, 4H, Ar-H), 7.54 (d, 2H, *J* = 8.2 Hz, Ar-H), 7.72 (d, 2H, *J* = 8.2 Hz, Ar-H), 7.98 (s, 1H, N=CH). ^13^C-NMR (DMSO-*d_6_*, 100 MHz): *δ* 161.26, 142.30, 138.88, 138.83, 133.08, 132.94, 131.23, 129.99, 129.28, 126.85, 120.84, 119.42, 82.96, 82.73, 74.84, 73.91, 21.15. MS *m/z* 301 (M + 1). ESI-HRMS calcd for C19H16N4^+^ ([M + H]^+^) 301.1448; found: 301.1452.

### Evaluation of antibacterial activity in vitro

Test bacteria were grown to mid-log phase in Mueller**–**Hinton broth (MHB) or Tryptone Soya Broth (TSB) and diluted 1000-fold in the same medium. Bacteria (10^5^ CFU/mL) were inoculated into MHB or TSB and dispensed at 0.2 ml/well into a 96-well microtiter plate. As positive controls, gatifloxacin, moxifloxacin, norfloxacin, oxacillin, and penicillin were used. Test compounds were prepared in DMSO, the final concentration of which did not exceed 0.05%. The minimum inhibitory concentration (MIC) was defined as the concentration of a test compound that completely inhibited bacterial growth after 24 h incubation at 37 °C. Bacterial growth was determined by measuring the absorption at 630 nm using a microplate reader. All experiments were carried out three times.

### Evaluation of bacterial resistance development

To evaluate the propensity for developing bacterial resistance, one of the compounds with high antibacterial activity (**3g**) was used. The initial MIC values of **3g** were determined against bacteria *Staphylococcus aureus* CMCC 25923 and *Escherichia coli* CMCC 44568, using norfloxacin and colistin, respectively, as antibiotic controls. Subsequently, serial passaging was initiated by transferring bacterial suspension grown at the sub-MIC of the compound/antibiotics (MIC/2) to a new plate and subjecting it to another assay to determine the (new) MIC. After 22 h incubation, cells grown at the sub-MIC of the test compound/antibiotics were once again transferred and the MIC determined. The process was repeated for 20 or 30 passages for *S. aureus* and *E. coli*, respectively. The MIC for **3g**, norfloxacin and colistin was plotted as a function of time in days (number of passages) to determine the propensity of bacterial resistance development^24^.

### Time-kill assay

Methicillin-resistant *S. aureus* ATCC 33591 grown in MHB was used to determine time-kill kinetics. Bacterial suspensions (10^5^ CFU/mL) containing test compounds (norflocaxin, compound **3g**) at final concentrations of 1 × MIC and 2 × MIC were incubated at 37 °C with shaking. Aliquots (10 μL) were removed from the cultures after 0, 0.5, 1, 2, 3, 4, 6, 8 and 12 h, serially diluted 1000-fold in nutrient solution, and plated onto sterile Mueller-Hinton agar medium. Plates were then incubated for 24 h at 37 °C, the number of CFU was counted, and the total bacterial log_10_ CFU/mL was determined.

### Evaluation of cytotoxicity activity *in vitro*

A lung cancer cell line (A549), gastric cancer cell line (SGC7901) and human hepatocytes (L02) were used to test the anticancer activity and cytotoxicity of the new compounds. 5-Fluorouracil (5-FU) was used as the positive control against cancer cell lines. The A549, SGC7901 and L02 cells were grown in Dulbecco’s modified Eagle’s medium (DMEM) supplemented with 10% foetal bovine serum (FBS) and antibiotics (100 U/mL penicillin-streptomycin). Cells at 80–90% confluence were split by trypsin (0.25% in PBS; pH 7.4), and the medium was changed at 24 h intervals. The cells were cultured at 37 °C in a 5% CO_2_ incubator. The cells were passaged three times, then approximately 1 × 10^4^ cells were seeded into each well of a 96-well plate and allowed to incubate to allow attachment of the cells to the surface. After 24 h, the medium was replaced with DMEM supplemented with 10% FBS containing various concentrations (0.1, 0.3, 1, 3, 10, and 30 μg/mL) of test compounds. Each concentration was tested in triplicate. After 48 h treatment, 20 µL of CCK-8 solution was added to each well and the optical density measured at 450 nm after 3 h using a microplate reader. The IC_50_ values were defined as the concentrations inhibiting 50% of cell growth.

### Docking studies

Molecular docking of compounds **3g** and **6e** to *Pseudomonas aeruginosa* LpxC (PDB code: 3P3E), *E. coli* LpxC (PDB code: 3P3G) and the *E. coli* FabH-CoA complex structure (PDB code: 1HNJ) was carried out using the DS–CDOCKER protocol implemented through the graphical user interface of the Discovery Studio software (version 2019). The structures of 3P3E, 3P3G, and 1HNJ were downloaded from Protein Data Bank. The three–dimensional structures of **3g** and **6e** were constructed using Chem3D Ultra 12.0 software (Chemical Structure Drawing Standard; CambridgeSoft Corporation, Waltham, MA, 2010) and was energetically minimised using the MMFF94 force field with 5000 iterations and a minimum RMS gradient of 0.10. For protein preparation, the hydrogen atoms were added and water and impurities were removed. The 3D structure of **3g** or **6e** was placed during the molecular docking procedure. Types of docking interactions of the proteins with tested compounds were analysed and ranked, and those with maximum binding energy were selected to analyse the interaction patterns.

## Results and discussion

### Chemistry

**Scheme 1.**
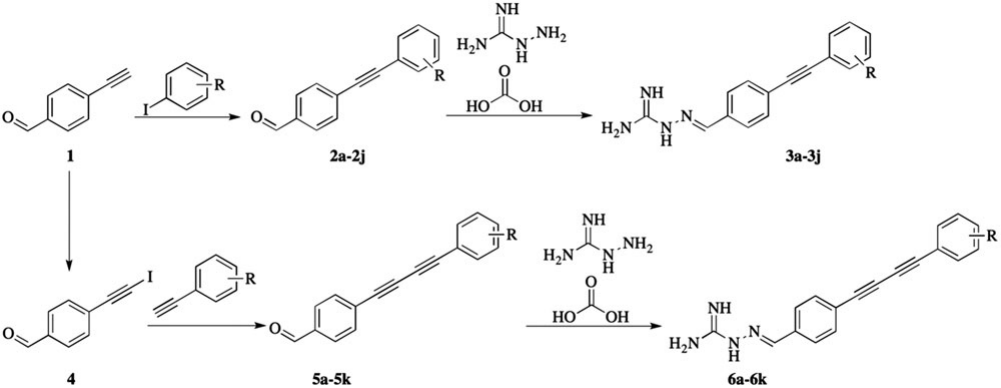
The synthetic route of aminoguanidine-linked alkynyl derivatives.

The synthesis of two series of aminoguanidine-linked alkynyl derivatives followed the general pathway outlined in Scheme 1 using 4-ethynylbenzaldehyde as starting material. The reaction of 4-ethynylbenzaldehyde with substituted-iodobenzenes in the presence of triethylamine (TEA), CuI and Pd(PPh_3_)Cl_2_ in tetrahydrofuran (THF) produced 4-(substituted-phenylethynyl)benzaldehyde (**2a–2j**) under the protection of nitrogen. The reaction of 4-ethynylbenzaldehyde with *N*-iodosuccinimide in the presence of AgNO_3_ in acetone produced 4-(iodoethynyl)benzaldehyde (**4**). The obtained 4-(iodoethynyl)benzaldehyde reacted with ethynylbenzenes in the presence of TEA, CuI and Pd(PPh_3_)Cl_2_ in THF yielding 4-((substituted-phenyl)buta-1,3-diyn-1-yl)benzaldehyde (**5a–5k**) under the protection of nitrogen. The target compounds **3a–3j** and **6a–6k** were prepared by the condensation of **2a–2j** or **5a–5k** with hydrazinecarboximidamide carbonate, respectively. Finally, the structures of the target compounds were characterised by ^1^H-NMR, ^13^C-NMR, and high-resolution mass spectrometry.

Spectroscopic analyses of all synthetic compounds fully supported their depicted structures. Taking compound **3a** as an example, the ^1^H-NMR spectrum yielded two singlets due to N-H of guanidyl at 5.65 and 6.04 ppm, which were assigned to two NH_2_ groups involved in the tautomerism of the guanidyl group. Peaks corresponding to the aromatic protons of terminal benzene ring were observed in the range 7.42–7.57 ppm. Two doublets (*J* = 8.2 Hz) due to aromatic protons of the *para*-substituted phenyl ring were observed at 7.50 and 7.72 ppm. The absorption peak of the C**–**H in imine was found at 7.99 ppm. The ^13^C-NMR spectra also identified 12 carbon nuclei in different chemical environments, which was also consistent with the structure of **3a**. Moreover, high-resolution mass spectrometry of **3a** displayed an [M + H]^+^ signal at *m*/*z* 263.1287, in agreement with its molecular weight of 263.1291.

### *In vitro* antibacterial activity

The evaluation of antibacterial activity of all compounds (**3a–3j** and **6a–6k**) was performed by a serial dilution method to determine the MIC against a panel of pathogens comprising five Gram-positive strains (*S. aureus* (CMCC(B) 26003 and CMCC 25923)*, Streptococcus mutans* BNCC 336931*, Enterococcus faecalis* CMCC 29212, and *Bacillus subtilis* CMCC 63501), four Gram-negative strains (*E. coli* (CMCC 25922 and CMCC 44568) and *P. aeruginosa* (CMCC 27853 and CMCC 10104)), as well as three methicillin-resistant clinical isolates (*S. aureus* ATCC 43300 and ATCC 33591, *P. aeruginosa* ATCC BAA-2111). The results are described in Tables 1 and 2. Gatifloxacin, moxifloxacin, norfloxacin, oxacillin, and penicillin were used as positive controls.

**Table 1.** Inhibitory activity (MIC, μg/mL) of compounds **3a–3j** and **6a–6k** against Gram-positive bacteria and Gram-negative bacteria.

Compound	R-	Gram-positive strains					Gram-negative strains			
		26003^a^	25923^b^	336931^c^	29212^d^	63501^e^	25922^f^	44568^g^	27853^h^	10104^i^
**3a**	H	1	4	4	1	2	4	4	16	4
**3b**	2-F	1	8	4	1	2	4	8	128	8
**3c**	3-F	1	4	2	1	1	2	4	16	2
**3d**	4-F	1	2	4	1	1	2	8	8	2
**3e**	2-Cl	0.5	2	2	0.5	1	2	8	>128	4
**3f**	3-Cl	1	1	2	0.5	0.5	2	4	>128	2
**3g**	4-Cl	0.5	0.5	0.5	0.5	0.25	2	2	>128	1
**3h**	2-Br	2	1	2	1	2	4	4	>128	8
**3i**	3-Br	1	2	1	1	1	2	2	>128	2
**3j**	4-Br	0.5	1	0.5	0.5	128	2	128	>128	128
**6a**	H	0.5	>128	0.5	0.5	0.5	128	128	>128	1
**6b**	2-F	0.25	1	0.5	0.5	4	8	128	>128	128
**6c**	3-F	128	128	0.5	0.5	2	2	>128	>128	4
**6d**	4-F	64	4	>128	128	>128	>128	>128	>128	>128
**6e**	2-Cl	0.25	128	1	0.5	4	8	64	>128	4
**6f**	3-Cl	1	8	0.5	0.5	4	4	32	>128	16
**6g**	4-Cl	>128	>128	>128	128	>128	128	128	>128	>128
**6h**	2-Br	1	32	1	0.5	2	8	128	>128	8
**6i**	3-Br	2	128	0.5	0.5	2	64	128	>128	128
**6j**	4-Br	128	>128	>128	128	>128	>128	>128	>128	>128
**6k**	3-CH_3_	1	32	0.5	0.5	2	4	128	>128	4
Gatifloxacin	–	0.125	0.125	1	1	2	0.125	0.125	2	2
Moxifloxacin	–	0.125	0.125	0.5	1	2	0.125	0.125	2	4
Norfloxacin	–	0.125	0.125	16	1	2	0.125	0.125	2	4
Oxacillin	–	0.125	0.125	0.125	128	>128	128	>128	>128	128
Penicillin	–	0.125	0.125	0.125	128	128	128	>128	>128	32

^a^ *Staphylococcus aureus* CMCC(B)26003.

^b^ *Staphyiococcus aureus* CMCC 25923.

^c^ *Streptococcus mutans* BNCC 336931.

^d^ *Enterococcus faecalis* CMCC 29212.

^e^ *Bacillus subtilis* CMCC 63501.

^f^ *Escherichia coli* CMCC 25922.

^g^ *Escherichia coli* CMCC 44568.

^h^ *Pseudomonas aeruginosa* CMCC 27853.

^i^ *Pseudomonas aeruginosa* CMCC 10104.

**Table 2.** Inhibitory activity (MIC, µg/mL) of compounds **3c–3e**, **3g**, **3i**, **3j**, **6a**, **6b** and **6e** against clinical isolates of multidrug-resistant strains.

Compound	R-	Multidrug-resistant Gram-positive strains		Multidrug-resistant Gram-negative strains
		43300^a^	33591^b^	BAA-2111^c^
**3c**	3-F	2	1	ND
**3d**	4-F	4	1	ND
**3e**	2-Cl	2	0.5	ND
**3g**	4-Cl	1	0.5	4
**3i**	3-Br	2	0.5	ND
**3j**	4-Br	2	0.5	ND
**6a**	H	4	0.5	>64
**6b**	2-F	8	1	ND
**6e**	2-Cl	8	0.5	ND
Gatifloxacin	–	0.5	0.25	1
Moxifloxacin	–	0.5	0.25	1
Norfloxacin	–	0.5	0.25	1
Oxacillin	–	64	8	ND
Penicillin	–	32	>32	ND

^a^ *Staphylococcus aureus* ATCC 43300.

^b^ *Staphylococcus aureus* ATCC 33591.

^c^ *Pseudomonas aeruginosa* ATCC BAA-2111.

ND: not detected.

In general, the inhibitory activity of the new target compounds against Gram-positive strains was better than that against Gram-negative strains. Compared with compounds **6a–6k** with a 1,4-diphenylbuta-1,3-diyne moiety, compounds **3a–3j** containing a 1,2-diphenylethyne moiety exhibited good to excellent antibacterial activity against all strains except *P. aeruginosa* CMCC 27853. *Pseudomonas aeruginosa* are able to rapidly develop resistance to multiple classes of antibiotics, making the treatment of infectious diseases becomes more challenging. The outer membrane porin OprD and the multidrug efflux pumps of *P. aeruginosa* represent the main barriers for drug entry into the cell. The decrease or loss of antibacterial activity of most compounds synthesized against *P. aeruginosa CMCC 27853* may be due to the above facts^25^.

All compounds in series **3a–3j** showed potent inhibitory effects against the five Gram-positive strains with MICs in the range 0.25–8 μg/mL, except compound **3j**, which showed inhibitory activity at 128 μg/mL against *B. subtilis* CMCC 63501. In general, compounds **3e**, **3f**, **3g**, **3j**, containing 2-Cl, 3-Cl, 4-Cl, and 4-Br, respectively, had greater inhibitory activity against Gram-positive strains. Compounds **3a–3j** were less active against the four Gram-negative strains, with MICs ranging from 1 µg/mL to more than 128 µg/mL. For *E. coli* CMCC 25922 and CMCC 44568, and *P. aeruginosa* CMCC 10104, the change of substituents had no obvious effect on the inhibitory activity, while for the *P. aeruginosa* CMCC 27853, the compounds with H, 3-F, 4-F groups had more potency than compounds with other substituents. Among the nine strains, compounds **3a–3j** presented the most potent inhibitory activity against *E. faecalis* CMCC 29212 with a MIC of 0.5 or 1 μg/mL, which is comparable to gatifloxacin, moxifloxacin and norfloxacin (MIC = 1 µg/mL) and is 256 or 128-fold more potent than oxacillin and penicillin (MIC = 128 µg/mL). Compound **3g** showed the most potent inhibitory activity against *B. subtilis* CMCC 63501 (MIC = 0.25 μg/mL).

Compounds in the series of **6a–6k** displayed different degrees of inhibitory activity (MICs ranging from 0.25 µg/mL to more than 128 µg/mL) against the five Gram-positive strains and two Gram-negative strains (*E. coli* CMCC 25922 and *P. aeruginosa* CMCC 10104), while all of them were insensitive towards *E. coli* CMCC 44568 and *P. aeruginosa* CMCC 27853. The structure**–**activity relationship showed that the *para*-position substituents reduce the antibacterial activity, giving compounds **6d**, **6g**, **6i** a MIC ≥128 against almost all of the tested strains. Compounds **6b** and **6e**, with F and Cl in the *ortho* position, showed the most potent inhibitory activity against *S. aureus* CMCC(B) 26003 (MIC = 0.25 μg/mL). Compound **6a**, without substituents on the terminal phenyl group, was more effective against *P. aeruginosa* CMCC 10104 than other compounds in this series.

Based upon their superior performance in the above assays, compounds **3c**, **3d**, **3e, 3g**, **3i**, **3j**, **6a**, **6b** and **6e** were further evaluated for their inhibitory activity against the growth of several clinical isolates of MDR bacterial strains (methicillin-resistant and/or oxacillin-resistant *S. aureus* ATCC 43300 and ATCC 33591 and multi-drug resistant *P. aeruginosa* ATCC BAA-2111). As shown in Table 2, compounds **3c**, **3d**, **3e, 3g**, **3i**, **3j**, **6a**, **6b** and **6e** presented good activities (MIC = 1–8 μg/mL) against MDR *S. aureus* ATCC *43300*, and showed the potent inhibitory activity against *S. aureus* ATCC 33591 (MIC = 0.5 or 1 μg/mL). They were slightly less active than gatifloxacin, moxifloxacin and norfloxacin (MIC = 0.25 µg/mL) but were more potent than oxacillin (MIC = 8 µg/mL) and penicillin (MIC > 32 µg/mL). Only compound **3g**, with Cl atom in the para position, showed moderate inhibitory activity against multi-drug resistant *P. aeruginosa* ATCC BAA-2111. It was also the most potent compound against growth of *S. aureus* ATCC 43300 and ATCC 33591.

### Propensity to develop bacterial resistance

**Figure 2.**
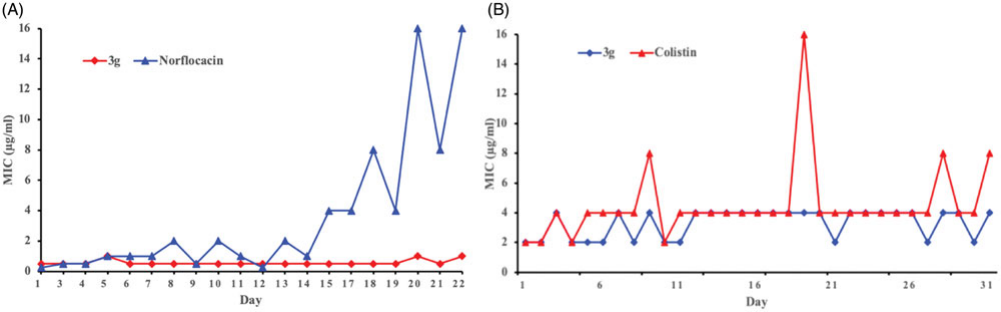
Propensity of the development of bacterial resistance towards compound **3g** by (A) *S. aureus* and (B) *E. coli*.

Bacterial resistance against most antibiotics presents a major problem of current times^26–29^. Therefore, it is crucial to evaluate the potential emergence of bacterial resistance against these biocides. The propensity for development of bacterial resistance of the synthesised compounds was evaluated by using the most active compound (**3g**) against both Gram-positive *S. aureus* and Gram-negative *E. coli*^30^^,^^31^. Norfloxacin, an antibiotic generally used to treat the Gram-positive infections, was used as a positive control for *S. aureus*, whereas colistin, a lipopeptide antibiotic active against Gram-negative bacteria, was used as a positive control for *E. coli*. These antibacterial agents were repeatedly challenged against bacteria at their sub-MIC values to allow bacteria to develop resistance. Resistance is usually defined as a > 4-fold increase in the original MIC^32^. Interestingly, little change in the MIC of compound **3g** was observed over 20 generations. In comparison, an approximately 32-fold and 4 or 8-fold increase in MIC was observed for norfloxacin and colistin, respectively (Figure 2). The above results indicated that bacteria do not develop resistance against this type of aminoguanidine within the experimental time period.

### Bactericidal time-kill kinetics

**Figure 3.**
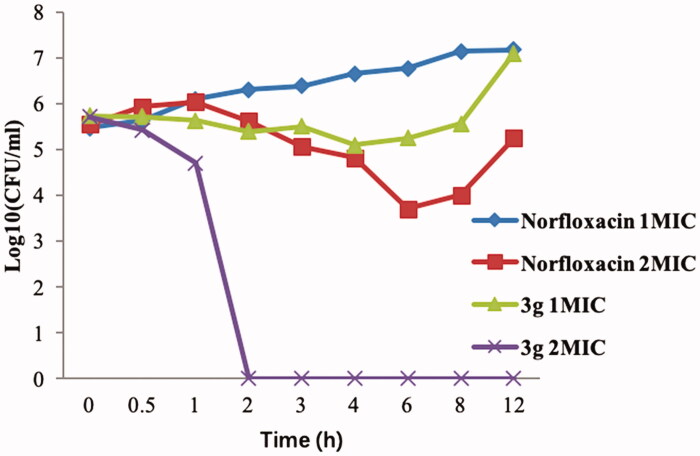
Bactericidal activities of compound **3g** and norfloxacin against MRSA.

To study the bactericidal activity of the promising compound **3g**, next we carried out *in vitro* time-kill assay against MRSA (starting bacterial concentration of 5.5 log10 CFU/mL) at two different concentrations (1 × MIC and 2 × MIC) using norfloxacin as a control (Figure 3). Compound **3g** was rapidly bactericidal at 2 × MIC (>5 log10 CFU/mL reduction) after 2 h and its bactericidal activity persisted for 12 h. In the case of norfloxacin, a concentration-dependent activity was seen at 1 × MIC to 2 × MIC, but its effects were bacteriostatic, not bactericidal (Figure 3). These results clearly demonstrate the superiority of compound **3g** over the commonly used antibiotic norfloxacin in killing MRSA bacteria.

### Molecular modelling

**Figure 4.**
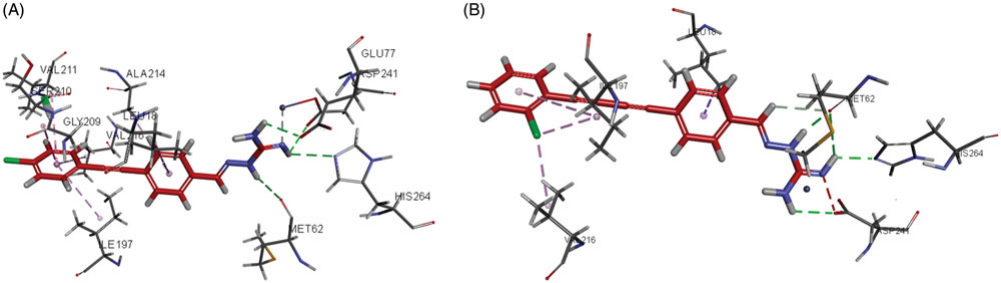
Interactions of compound **3g** and **6e** with *P. aeruginosa* LpxC (A for **3g**; B for **6e**).

LpxC is an essential enzyme in the lipid A biosynthetic pathway. Developing novel LpxC inhibitors has been an important approach to obtain new antibacterial drugs targeting Gram-negative pathogens. To gain insight into the molecular interactions of these compounds with LpxC, the co-crystal structures of **3g** and **6e** complexed with *P. aeruginosa* LpxC and *E. Coli* LpxC were obtained. Generally, the 1,2-diphenylethyne group in the series of **3a–3j** and 1,4-diphenylbuta-1,3-diyne moiety in the series of **6a–6k** functionally interacted with many of the hydrophobic residues in the lipophilic tunnel. The aminoguanidine was responsible for forming hydrogen bonds with several amino acid residues. The aminoguanidine of compound **3g** bound to the active site Zn^2+^ ion of *P. aeruginosa* LpxC (Figure 4(A)) and formed hydrogen bonds with ASP241, HIS264, MET62, and GLU77 that line this polar region. In the interaction of *P. aeruginosa* LpxC with compound **6e** (Figure 4(B)), the phenyl ring of 1,4-diphenylbuta-1,3-diyne moiety showed interactions with critical amino acid residues LEU18, ILE197, and VAL216 *via* aromatic stacking and hydrophobic intermolecular forces. The guanidine group of compound **6e** acted as a hydrogen bond donor in the interaction with the carbonyl group of ASP241, HIS264 and MET62.

**Figure 5.**
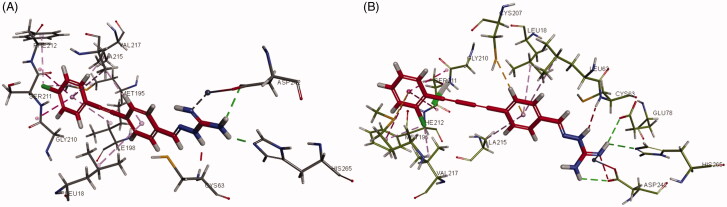
Interactions of (A) compound **3g** and (B) **6e** with *E. coli* LpxC.

Interactions of compounds **3g** and **6e** with *E. coli* LpxC are shown in Figure 5(A,B), respectively. The aminoguanidine of compound **3g** was bound to the active site Zn^2+^ ion and forms hydrogen bond interactions with ASP242 and HIS265. The 1,2-diphenylethyne group was responsible for forming various aromatic stacking interactions and hydrophobic intermolecular forces with LEU18, ALA215, VAL217, GLY210, SER211, ALA215, and ILE198. In addition, the Cl atom attached to the terminal phenyl group showed hydrophobic interactions with amino acid residues PHE212 and MET195 that increased the binding force with *E. coli* LpxC. The aminoguanidine of compound **6e** was also bound to the zinc atom of *E. coli* LpxC, as well as the amino acid residues ASP242, GLU78, and HIS265 *via* hydrogen bond interaction. Aromatic stacking interactions and hydrophobic force interactions were additionally formed between the hydrophobic 1,4-diphenylbuta-1,3-diyne moiety of **6e** and LEU18, CYS207, ALA215, LEU62, MET195, GLY210, SER211, PHE212, and VAL217.

**Figure 6.**
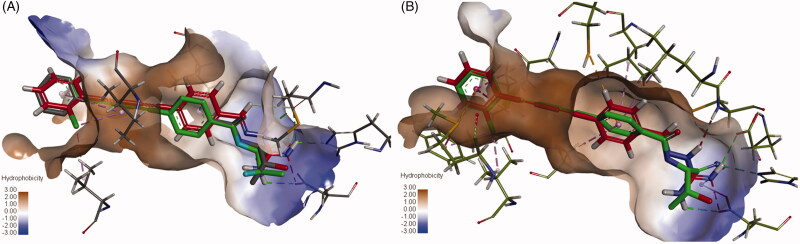
Overlay of **6e** and **LPC-009** binding to (A) *P. aeruginosa* LpxC and (B) *E. coli* LpxC.

For comparison, the interactions of compound **6e** with *P. aeruginosa* LpxC and *E. coli* LpxC were superimposed on the co-crystallised LPC-009. As seen in Figure 6, compound **6e** (red) showed similar interactions as LPC-009 (green). This suggests that binding of **6e** to LpxC may be, at least in part, responsible for its antibacterial activity.

It is likely that targets other than LpxC might be involved in the broad-spectrum antibacterial activity of the synthesised compounds against Gram-positive and Gram-negative bacteria. The FabH receptor is a condensing enzyme that plays key roles in fatty acid biosynthesis^33^. The potential interaction of some hydrazine compounds with FabH prompted us to investigate the molecular interactions of the representative compounds **3g** and **6e** with FabH receptor (PDB ID: 1HNJ)^20^^,^^34^^,^^35^.

**Figure 7.**
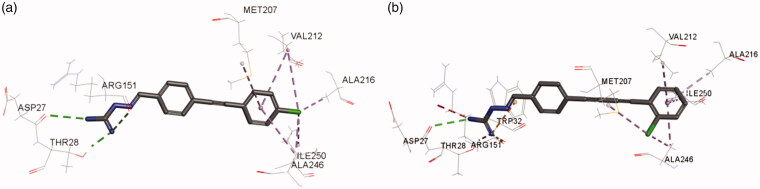
Interactions of compound (A) **3g** and (B) **6e** with *E. coli* FabH.

As shown in Figure 7(A), residues including ASP27, THR28, ARG151, VAl212, ALA216, ILE250, and ALA246, were involved in the binding of **3g** to the active site of *E. coli* FabH. The C = N and NH groups of compound **3g** were involved in the interaction with ASP27, THR28, ARG151 *via* hydrogen bonding, while the terminal phenyl group and its attached chlorine atom formed hydrophobic interactions with VAl212, ALA216, ILE250, and ALA246. The binding of **6e** with *E. coli* FabH resembled that of **3g**. As shown in Figure 6(B), residues ASP27, THR28, ARG151, TRP32, VAl212, ALA216, ILE250, and ALA246, were involved in the interactions with **6e** in *E. coli* FabH enzyme.

### Anticancer activity

The cytotoxic effects of compounds **3e**, **3f**, **3g**, **3i**, **6a**, **6b**, **6e** and **6k** were evaluated using two human cancer cell lines (A549and SGC7901) and one human normal cell line (L02). The IC_50_ values of the tested compounds and 5-FU are shown in Table 3. All the tested compounds showed excellent activity against the investigated cancer cells (IC_50_ = 0.30–4.57 µg/mL); however, no correlation between the substituents and the cytotoxic activity could be identifies. The highest activity against A549 cells was exhibited by compound **6b** (IC_50_ = 2.22 µg/mL), although it is not comparable to that of 5-FU, which exhibited IC_50_ of 0.88 µg/mL. The highest activity against SGC7901 cells was exhibited by compound **3f** (IC_50_ = 0.30 µg/mL), followed by compounds **3e** and **6k** (IC_50_ = 0.45, 0.55 µg/mL, respectively). All the tested compounds showed higher cytotoxic activity against SGC7901 cells than 5-FU. Compounds **3e**, **3f**, **3g**, **3i**, **6a**, **6b**, **6e** and **6k** showed IC_50_ values in the range of 10.25–20.85 µg/mL against the normal cell line L02, comparing to the IC_50_ value of 8.44 µg/mL of 5-FU. This result indicated that these compounds have low toxicity towards normal cells in comparison to cancer cells, suggesting potential for a good therapeutic index.

**Table 3.** The Inhibitory activity (IC_50_, µg/mL) of compounds **3e**, **3f**, **3g**, **3i**, **6a**, **6b**, **6e and 6k** against cancer cell lines A549 and SGC7901 and normal cell lines L02.

Compound	R-	A549	SGC7901	L02
**3e**	2-Cl	4.57	0.45	12.43
**3f**	3-Cl	2.55	0.30	10.25
**3g**	4-Cl	4.42	1.01	20.85
**3i**	3-Br	4.03	1.26	19.63
**6a**	H	2.49	1.63	13.36
**6b**	2-F	2.22	1.37	17.98
**6e**	2-Cl	3.62	1.48	14.80
**6k**	4-Br	3.58	0.55	14.77
**5-Fluorouracil**	–	0.88	2.56	8.44

## Conclusion

In the present work, a series of novel aminoguanidines containing an alkynyl moiety were synthesised and characterised. The antibacterial and anticancer activities of these compounds were screened, with Gram-positive bacteria being more susceptible than Gram-negative ones. The aminoguanidine derivatives (**3a–3j**), having a 1,2-diphenylethyne, exhibited greater antibacterial activity than **6a–6j** with a 1,4-diphenylbuta-1,3-diyne moiety. Compounds **3a–3j** showed potent inhibitory activity against the selected bacterial strains with MIC values in the range of 0.25–8 μg/mL, including the multidrug resistant strains. Among them, compound **3g** was the most promising, having superior activity to oxacillin and penicillin against the tested MDR strains. Resistance of the tested bacteria towards **3g** was not easily developed and this compound was rapidly bactericidal. Furthermore, **3 g** exhibited significant anticancer activity against lung (A549) and gastric cancer cells (SGC7901), with IC_50_ values of 4.42 and 1.01 µg/mL, respectively, and low-toxicity towards normal cells. To understand the binding pattern, molecular docking of representative compounds **3g** and **6e** was performed, demonstrating that they bind strongly to the LpxC enzyme and FabH enzyme. These findings indicate that compounds containing the aminoguanidine moiety are promising candidates for the development of new antibacterial and anticancer agents.

## References

[1] Magiorakos AP, Srinivasan A, Carey RB, et al. Multidrug-resistant, extensively drug-resistant and pan drug-resistant bacteria: an international expert proposal for interim standard definitions for acquired resistance. Clin Microbiol Infect 2012;18:268–81. 21793988 10.1111/j.1469-0691.2011.03570.x

[2] Magiorakos AP, Suetens C, Monnet DL, et al. The rise of carbapenem resistance in Europe: just the tip of the iceberg?. Antimicrob Resist Infect Control 2013;2:6. 23410479 10.1186/2047-2994-2-6 PMC3691711

[3] Freire-Moran L, Aronsson B, Manz C, et al. Critical shortage of new antibiotics in development against multidrug-resistant bacteria-Time to react is now. Drug Resist Updat 2011;14:118–24. 21435939 10.1016/j.drup.2011.02.003

[4] Payne DJ, Gwynn MN, Holmes DJ, et al. Drugs for bad bugs: confronting the challenges of antibacterial discovery. Nat Rev Drug Discov 2007;6:29–40. 17159923 10.1038/nrd2201

[5] Whitfield C, Trent MS. Biosynthesis and export of bacterial lipopolysaccharides. Annu Rev Biochem 2014;83:99–128. 24580642 10.1146/annurev-biochem-060713-035600

[6] Liu F, Ma S. Recent process in the inhibitors of UDP-3-O-(R-3-hydroxyacyl)-nacetylglucosamine deacetylase (LpxC) against Gram-negative bacteria. Mini Rev Med Chem 2018;18:310–23. 27739357 10.2174/1389557516666161013120253

[7] Zhang J, Zhang L, Li X, Xu W. UDP-3-O-(R-3-hydroxymyristoyl)-N-acetylglucosamine deacetylase (LpxC) inhibitors: a new class of antibacterial agents. Curr Med Chem 2012;19:2038–50. 22414079 10.2174/092986712800167374

[8] Erwin AL. Antibacterial drug discovery targeting the lipopolysaccharide biosynthetic enzyme LpxC. Cold Spring Harb Perspect Med 2016;6:a025304. 27235477 10.1101/cshperspect.a025304 PMC4930914

[9] Coggins BE, Li X, McClerren AL, et al. Structure of the LpxC deacetylase with a bound substrate-analog inhibitor. Nat Struct Biol 2003;10:645–51. 12833153 10.1038/nsb948 PMC6783277

[10] Clayton GM, Klein DJ, Rickert KW, et al. Structure of the bacterial deacetylase LpxC bound to the nucleotide reaction product reveals mechanisms of oxyanion stabilization and proton transfer. J Biol Chem 2013;288:34073–80. 24108127 10.1074/jbc.M113.513028 PMC3837148

[11] Onishi HR, Pelak BA, Gerckens LS, et al. Antibacterial agents that inhibit lipid A biosynthesis. Science 1996;274:980–2. 8875939 10.1126/science.274.5289.980

[12] Lee PS, Lapointe G, Madera AM, et al. Application of virtual screening to the identification of new LpxC inhibitor chemotypes, oxazolidinone and isoxazoline. J Med Chem 2018;61:9360–70. 30226381 10.1021/acs.jmedchem.8b01287

[13] Hale MR, Hill P, Lahiri S, et al. Exploring the UDP pocket of LpxC through amino acid analogs. Bioorg Med Chem Lett 2013;23:2362–7. 23499237 10.1016/j.bmcl.2013.02.055

[14] Mansoor UF, Vitharana D, Reddy PA, et al. Design and synthesis of potent Gram-negative specific LpxC inhibitors. Bioorg Med Chem Lett 2011;21:1155–61. 21273067 10.1016/j.bmcl.2010.12.111

[15] Liang X, Lee CJ, Chen X, et al. Syntheses, structures and antibiotic activities of LpxC inhibitors based on the diacetylene scaffold. Bioorg Med Chem 2011;19:852–60. 21194954 10.1016/j.bmc.2010.12.017 PMC3035996

[16] Lee CJ, Liang X, Chen X, et al. Species-specific and inhibitor-dependent conformations of LpxC: implications for antibiotic design. Chem Biol 2011;18:38–47. 21167751 10.1016/j.chembiol.2010.11.011 PMC3149848

[17] Liang X, Lee CJ, Zhao J, et al. Synthesis, structure, and antibiotic activity of aryl-substituted LpxC inhibitors. J Med Chem 2013;56:6954–66. 23914798 10.1021/jm4007774 PMC3941642

[18] Gao ZM, Wang TT, LI SZ, et al. Synthesis and antibacterial activity evaluation of (2-chloroquinolin-3-yl)methyleneamino guanidine derivatives. Chin J Org Chem 2016;36:2484–8.

[19] Yu HH, Zhou SC, Guo TT, et al. Synthesis and antimicrobial activity evaluation of aminoguanidine derivatives containing a biphenyl moiety. Chin J Org Chem 2019;39:1497–502

[20] Song MX, Wang SB, Wang ZT, et al. Synthesis, antimicrobial and cytotoxic activities, and molecular docking studies of N-arylsulfonylindoles containing an aminoguanidine, a semicarbazide, and a thiosemicarbazide moiety. Eur J Med Chem 2019;166:108–18. 30685534 10.1016/j.ejmech.2019.01.038

[21] Sidoryk K, Świtalska M, Rózga P, et al. An efficient synthesis of indolo[2,3-b]quinoline guanidine derivatives with their in vitro and in vivo study. Med Chem Res 2017;26:3354–66. 29170613 10.1007/s00044-017-2028-1 PMC5676820

[22] Wei ZY, Chi KQ, Yu ZK, et al. Synthesis and biological evaluation of chalcone derivatives containing aminoguanidine or acylhydrazone moieties. Bioorg Med Chem Lett 2016;26:5920–5. 27843112 10.1016/j.bmcl.2016.11.001

[23] Osowska K, Lis T, Szafert S. Protection/deprotection-free syntheses and structural analysis of (*Keto-aryl)diynes. Eur J Org Chem 2008;27:4598–606.

[24] Qiu X, Janson CA, Smith WW, et al. Refined structures of β-ketoacyl-acyl carrier protein synthase III. J Mol Biol 2001;307:341–56. 11243824 10.1006/jmbi.2000.4457

[25] Lister PD, Wolter DJ, Hanson ND. Antibacterial-resistant *Pseudomonas aeruginosa*: clinical impact and complex regulation of chromosomally encoded resistance mechanisms. Clin Microbiol Rev 2009;22:582–610. 19822890 10.1128/CMR.00040-09 PMC2772362

[26] Niu Y, Wang RE, Wu H, Cai J. Recent development of small antimicrobial peptidomimetics. Future Med Chem 2012;4:1853–62. 23043481 10.4155/fmc.12.111

[27] Taubes G. The bacteria fight back. Science 2008;321:356–61. 18635788 10.1126/science.321.5887.356

[28] Walsh C. Molecular mechanisms that confer antibacterial drug resistance. Nature 2000;406:775–81. 10963607 10.1038/35021219

[29] Alekshun MN, Levy SB. Molecular mechanisms of antibacterial multidrug resistance. Cell 2007;128:1037–50. 17382878 10.1016/j.cell.2007.03.004

[30] Makovitzki A, Avrahami D, Shai Y. Ultrashort antibacterial and antifungal lipopeptides. Proc Natl Acad Sci U S A 2006;103:15997–6002. 17038500 10.1073/pnas.0606129103 PMC1635116

[31] Hoque J, Akkapeddi P, Yadav V, et al. Broad spectrum antibacterial and antifungal polymeric paint materials: synthesis, structure-activity relationship, and membrane-active mode of action. ACS Appl Mater Interfaces 2015;7:1804–15. 25541751 10.1021/am507482y

[32] Kosowska-Shick K, Clark C, Pankuch GA, et al. Activity of telavancin against staphylococci and enterococci determined by MIC and resistance selection studies. Antimicrob Agents Chemother 2009;53:4217–24. 19620338 10.1128/AAC.00742-09 PMC2764208

[33] Heath RJ, Rock CO. Inhibition of beta-ketoacyl-acyl carrier protein synthase III (FabH) by acyl-acyl carrier protein in *Escherichia coli*. J Biol Chem 1996;271:10996–1000. 8631920 10.1074/jbc.271.18.10996

[34] Chu W-C, Bai P-Y, Yang Z-Q, et al. Synthesis and antibacterial evaluation of novel cationic chalcone derivatives possessing broad spectrum antibacterial activity. Eur J Med Chem 2018;143:905–21. 29227931 10.1016/j.ejmech.2017.12.009

[35] Zhang HJ, Qin X, Liu K, et al. Synthesis, antibacterial activities and molecular docking studies of Schiff bases derived from N-(2/4-benzaldehyde-amino) phenyl-N′-phenyl-thiourea. Bioorg Med Chem 2011;19:5708–15. 21872479 10.1016/j.bmc.2011.06.077

